# Selected Reaction Monitoring (SRM) Analysis of Epidermal Growth Factor Receptor (EGFR) in Formalin Fixed Tumor Tissue

**DOI:** 10.1186/1559-0275-9-5

**Published:** 2012-05-03

**Authors:** Todd Hembrough, Sheeno Thyparambil, Wei-Li Liao, Marlene M Darfler, Joseph Abdo, Kathleen M Bengali, Paul Taylor, Jiefei Tong, Humberto Lara-Guerra, Thomas K Waddell, Michael F Moran, Ming-Sound Tsao, David B Krizman, Jon Burrows

**Affiliations:** 1 Onco Plex Diagnostics Inc. 9620 Medical Center Drive, Rockville, Maryland, 20850, USA; 2 Program in Molecular Structure and Function, Hospital For Sick Children, 555 University Avenue, Toronto, ON, M5G 1X8, Canada; 3 Division of Thoracic Surgery, Toronto General Hospital Research Institute, University Health Network, 610 University Avenue, Toronto, ON, M5G 2 M9, Canada; 4 Ontario Cancer Institute, Princess Margaret Hospital, University Health Network, 610 University Avenue, Toronto, ON, M5G 2 M9, Canada; 5 Molecular Genetics, Banting and Best Department of Medical Research, Institute of Medical Science, Toronto, ON, M5S 1A8, Canada; 6 Department of Surgery, 2374 Medical Sciences Building, 1 King's College Circle, University of Toronto, Toronto, ON, M5S 1A8, Canada; 7 Laboratory Medicine and Pathology, Medical Biophysics Institute of Medical Science, Toronto, ON, M5S 1A8, Canada; 8 Department of Surgery, 2374 Medical Sciences Building, 1 King's College Circle, University of Toronto, Toronto, ON, M5S 1A8, Canada; 9 Onco Plex Diagnostics Inc., 9620 Medical Center Drive, Rockville, 20850, Maryland, USA

**Keywords:** Formalin fixed, FFPE, EGFR, Gefitinib, Targeted therapy, Patient tissue, Quantitative, Personalized medicine, Molecular diagnostics, Non-small cell lung cancer

## Abstract

**Background:**

Analysis of key therapeutic targets such as epidermal growth factor receptor (EGFR) in clinical tissue samples is typically done by immunohistochemistry (IHC) and is only subjectively quantitative through a narrow dynamic range. The development of a standardized, highly-sensitive, linear, and quantitative assay for EGFR for use in patient tumor tissue carries high potential for identifying those patients most likely to benefit from EGFR-targeted therapies.

**Methods:**

A mass spectrometry-based Selected Reaction Monitoring (SRM) assay for the EGFR protein (EGFR-SRM) was developed utilizing the Liquid Tissue®-SRM technology platform. Tissue culture cells (n = 4) were analyzed by enzyme-linked immunosorbent assay (ELISA) to establish quantitative EGFR levels. Matching formalin fixed cultures were analyzed by the EGFR-SRM assay and benchmarked against immunoassay of the non-fixed cultured cells. Xenograft human tumor tissue (n = 10) of non-small cell lung cancer (NSCLC) origin and NSCLC patient tumor tissue samples (n = 23) were microdissected and the EGFR-SRM assay performed on Liquid Tissue lysates prepared from microdissected tissue. Quantitative curves and linear regression curves for correlation between immunoassay and SRM methodology were developed in Excel.

**Results:**

The assay was developed for quantitation of a single EGFR tryptic peptide for use in FFPE patient tissue with absolute specificity to uniquely distinguish EGFR from all other proteins including the receptor tyrosine kinases, IGF-1R, cMet, Her2, Her3, and Her4. The assay was analytically validated against a collection of tissue culture cell lines where SRM analysis of the formalin fixed cells accurately reflects EGFR protein levels in matching non-formalin fixed cultures as established by ELISA sandwich immunoassay (R^2^ = 0.9991). The SRM assay was applied to a collection of FFPE NSCLC xenograft tumors where SRM data range from 305amol/μg to 12,860amol/μg and are consistent with EGFR protein levels in these tumors as previously-reported by western blot and SRM analysis of the matched frozen tissue. In addition, the SRM assay was applied to a collection of histologically-characterized FFPE NSCLC patient tumor tissue where EGFR levels were quantitated from not detected (ND) to 670amol/μg.

**Conclusions:**

This report describes and evaluates the performance of a robust and reproducible SRM assay designed for measuring EGFR directly in FFPE patient tumor tissue with accuracy at extremely low (attomolar) levels. This assay can be used as part of a complementary or companion diagnostic strategy to support novel therapies currently under development and demonstrates the potential to identify candidates for EGFR-inhibitor therapy, predict treatment outcome, and reveal mechanisms of therapeutic resistance.

## Introduction

Many human cancers are associated with over-expressed epidermal growth factor receptor (EGFR) [1]. EGFR is a validated, potential therapeutic target in many human cancers including colorectal, ovarian, head and neck, bladder, lung, pancreatic, and breast. Over-expression and/or activation of EGFR by ligand are associated with activation of several cytoplasmic proteins that induce a signal that stimulates intracellular responses and activation of signaling pathway proteins in the RAS/RAF/MAPK, STAT and PI3K/AKT pathways, which together modulate cellular proliferation, adhesion, angiogenesis, migration, and survival [2,3]. Cellular EGFR activity is a product of complex factors including expression level, ligands, mutation, dimerization-regulated conformation. Analysis of EGFR at the protein level, however, in clinical tissue samples (with respect to patient tumor profiling and targeted therapy) is typically done by immunohistochemistry (IHC) that is only subjectively quantitative through a narrow dynamic range. EGFR-directed antibodies (cetuximab and panitumumab) and small molecule inhibitors (gefitinib and erlotinib) provide positive therapeutic effects in several types of cancer, including non-small cell lung cancer (NSCLC). Clinical data show that EGFR expression levels as currently determined by IHC historically do not predict clinical outcome in response to anti-EGFR targeted therapy [4,5]. However, recent reports suggest EGFR protein levels may provide some predictive response data to gefitinib and cetuximab in NSCLC patients but that the choice of diagnostic antibody and IHC methodology is paramount to predicting response and outcome to specific therapies [6,7]. These reports suggest that measuring EGFR in patient tissue may indeed correlate to therapeutic outcome, yet it is clear that application of alternative, robust, reproducible, and scalable technologies for conclusive objective quantitation of the EGFR protein is required to predict and improve therapeutic outcomes through more optimal patient selection, monitoring, and dosing.

Mass spectrometry (MS) has emerged as a method to quantitatively analyze proteins in biological samples, and application of MS to patient-derived tissue could have a large, positive impact on patient stratification and targeted cancer therapeutics [8-12]. The Liquid Tissue-SRM technology platform is based on application of mass spectrometry to solubilized protein lysates from formalin fixed, paraffin embedded (FFPE) patient-derived tissue [13-20]. This report demonstrates development of a robust, reproducible Liquid Tissue-SRM assay capable of conclusively measuring EGFR protein levels in Liquid Tissue lysates prepared from formalin fixed samples including patient-derived FFPE tumor tissue. Clinical application of this assay could provide for patient selection to optimize EGFR-targeted therapy strategies, predict patient outcome, and determine the potential for therapeutic resistance to other receptor tyrosine kinase inhibitor molecules.

## Materials and Methods

### Cell lines

Four human cell lines, A431 (skin), HT29 (colon), MDA-MB-231 (breast), and MCF7 (breast) were utilized for this study. A431 and MDA-MB-231 lines were maintained in Dulbecco’s Modified Eagle Medium (DMEM), HT29 cells were maintained in McCoy’s5A medium, and MCF7 cells were maintained in DMEM-F12. All media were supplemented with 10 % fetal bovine serum (FBS) and antibiotics. Each cell line was grown in sufficient quantity so that preparations of each cell line could be processed in parallel for both ELISA and SRM analysis.

Formalin fixed paraffin embedded (FFPE) culture cells were prepared by pelleting cell suspensions, overlaying the pellet with 10 % neutral-buffered formalin (NBF), and allowing the cells to fix for 18 to 24 hours at 4°C. The 10 % formalin was removed and the pellet was washed with water and then transferred into 70 % ethanol. Embedding in paraffin and sectioning of cells prepared on microscope slides were done using standard histology methods.

### Tissue and tissue microdissection

Ten (10) human xenograft FFPE tumor blocks of non-small cell lung carcinoma (NSCLC) origin were obtained from a collection of xenograft tumors previously described [21]. Twenty-three (23) FFPE NSCLC patient tissue blocks were obtained without patient identifiers from the University of Toronto Princess Margaret Hospital/University Health Network with informed consent of all participating subjects under strict Institutional Review Board (IRB) standards and Ethical Committee approval. All patients were participants in a phase II clinical trial where they received neoadjuvant treatment with the EGFR-inhibitor gefitinib prior to surgical resection of their tumor [22]. Histological analysis indicates a mixture of adenocarcinoma, squamous cell carcinoma, and one large cell carcinoma in the cohort. Multiple ten micron (10 μm) thick sections from each of the FFPE cell line blocks, FFPE NSCLC xenograft tumor blocks, and the FFPE NSCLC patient tissue blocks were cut onto DIRECTOR® slides for tissue microdissection according to manufacturer’s recommendations (Onco Plex Diagnostics Inc. Rockville, MD).

DIRECTOR slides with sectioned FFPE cultured cells, xenograft tumor tissue, and patient tumor tissue were deparaffinized and stained with hematoxylin using standard histological methods prior to dissection. Microdissection was performed on a Leica LMD6000 dissection scope according to manufacturer’s recommendations (Leica, Wetzlar, Germany). A total area of 12 mm^2^ consisting of approximately 45,000 formalin fixed cultured cells or tissue-derived cancerous cells were transferred from the FFPE sections via laser dissection directly into the dry cap of a 0.5 ml tube. Once the entire 12 mm^2^ area was transferred to the dry cap, 20ɥl of 100 % acetonitrile (ACN) was added to the cap and the cell/tissue material was transferred to the bottom of the tube by a brief centrifugation. The ACN was removed by speedvac centrifugation at 35°C for 6 min. The dried dissection pellet was stored at −20°C.

### Sample preparation and immunoassay

Protein lysates of fresh, unfixed cells for each culture cell line were prepared for analysis by enzyme-linked immunosorbent assay (ELISA) using the manufacturer-provided cell lysis buffer and protocol according to manufacturer’s recommendations (Millipore, Billerica, MA). Adherent cells were washed twice in the flask with ice-cold PBS and all the PBS was drained. Ice-cold lysis buffer (modified RIPA buffer) was added to the cells (1 mL per 10^7^ cells/100 mm dish/150 cm^2^ flask). Cells were scraped off the flask with a plastic cell scraper and the cell suspension was transferred to a centrifuge tube where the suspension was gently rocked on an orbital shaker 15 min to lyse cells. The lysate was centrifuged at 14,000 g for 15 min and the supernatant was transferred to a fresh centrifuge tube. The pellet was discarded. Total protein content was determined by a Micro BCA assay according to manufacturer’s recommendations (Thermo Fisher Scientific Inc, Rockford, IL). The lysate was divided into aliquots and stored at −20°C. EGFR levels were determined by ELISA-based sandwich immunoassay utilizing an ELISA kit according to manufacturer’s recommendations (Millipore, Billerica, MA). ELISA immunoassay was performed on each protein lysate in triplicate and data collected on a μQuant plate reader (Bio-Tek Instruments, Winooski, VT).

### SRM assay development

Recombinant EGFR protein was obtained and analyzed on an Orbitrap mass spectrometer (Thermo Scientific, San Jose, CA) equipped with a nanoAcquityLC system (Waters, Milford, MA) and on a TSQVantage triplequadrupole mass spectrometer (Thermo Scientific, San Jose, CA) equipped with a nanoAcquityLC to evaluate all tryptic peptides in order to identify candidate SRM peptides. Liquid Tissue lysates prepared from FFPE A431 cells were analyzed on the TSQVantage system. Software programs Pinpoint1.0, Xcalibur2.1 (Thermo Scientific, San Jose, CA) were used to identify optimal tryptic peptides based on reproducible peak heights, retention times, chromatographic ion intensities, and distinctive/reproducible transition ion ratios. Methionine and cysteine-containing peptides were excluded due to the existence of various oxidation entities. Peptides with glycosylation motifs were also excluded. The uniqueness of observed peptides was verified by performing a peptide sequence search using the BLASTP function of the BLAST search engine (http://blast.ncbi.nlm.nih.gov/Blast.cgi) and a single peptide unique to EGFR was chosen which spans residues 98–108 of the EGFR extracellular domain. Unlabeled (IPLENLQIIR) and isotopically-labeled (IPLEN[13CN15]LQIIR) versions of this peptide were synthesized to develop and perform the assay (Thermo Scientific, San Jose, CA). SRM transitions used for the quantification of EGFR using unlabeled peptide were 604.872/756.472 (y6^+1^), 885.515 (y7^+1^), 998.599 (y8^+1^) (Q1/Q3); and transitions used for quantitation of EGFR using isotopically labeled peptide 608.36/763.489 (y6^+1^), 892.532 (y7^+1^), 1005.616 (y8^+1^) (Q1/Q3).

A standard curve was developed by serial dilution of the light peptide against a constant concentration of labeled peptide in a non-human complex tryptic peptide mixture from *Pyrococcus furiosus* (*P. furiosus*) (Agilent Technologies Inc, Santa Clara, CA) on the TSQVantage system utilizing the following conditions; Q1(FWHM);0.2, Q2(FWHM):0.7, dwell time;10 ms. Due to non-availability of a “standard tissue matrix” for development of a standard curve, the *P. furiosus* matrix was used as a simulation of the matrix that exists in the tissue environment. Data was analyzed by Pinpoint1.0 to determine the limit of detection (LOD) and limit of quantitation (LOQ). The LOD was determined by identifying the lowest concentration in the standard curve where the transition ion ratios and co-elution profile of the unlabeled synthetic peptide were similar to labeled synthetic peptide. Additionally, a signal to noise ratio >3 and a CV from triplicate measurements ≤25 % were used. The LOQ was determined by identifying the next highest concentration of the standard curve above the LOD with a CV ≤ 25 % and signal to noise ratio >10.

### Sample preparation and SRM analysis

Liquid Tissue lysates were prepared for SRM analysis using the Liquid Tissue preparation protocol and reagents according to manufacturer’s recommendations (Onco Plex Diagnostics Inc. Rockville, MD) for each of the microdissected FFPE cell lines and FFPE tissue samples. Briefly, dried microdissection pellets were suspended in 30 μl of Liquid Tissue buffer and heated at 95°C for 90 min with gentle agitation every 20 min followed by cooling on ice at which point 1.5 μl of trypsin (1 μg/μl) was added to each tube. Tubes were incubated at 37°C for 16 to 18 hours. Liquid Tissue preparations were stored at −20°C. Total protein content for each Liquid Tissue lysate was determined by a Micro BCA assay (Thermo Fisher Scientific Inc, Rockford, IL).

For analysis of Liquid Tissue lysates from both microdissected FFPE cultured cells and FFPE microdissected patient tissue, 5 μg of Liquid Tissue lysate was diluted to 45 μl with 0.1 % formic acid, and then 5 μl of the isotopically labeled internal standard peptide (5fmol/μl) was added. Following a centrifugation step at 10,000 x g for 10 min, 45 μl of each supernatant was placed in the TSQ Vantage system’s autosampler. Ten microliters (10 μl) of each sample, containing 1 μg total protein and 5fmol of isotopically labeled internal standard EGFR peptide, were directly injected into the system at a loading flow rate of 5 μl/min. Liquid Tissue lysates were directly injected into the LC system with no processing because the proprietary buffer contains no components that may interfere with chromatography or suppress ionization. Direct injection of Liquid Tissue lysates has been demonstrated in multiple reports and across different mass spectrometry platforms [14-20].

Peptide separations were performed on an nanoAcquityLC system (Waters, Milford, MA) or EASY-nLC II (Thermo Scientific, San Jose, CA) with a PicoFrit (100 μm ID/10μm tip ID, New Objective) column self-packed to a bed length of 12 cm with Jupiter Proteo 90Å C12, 4 μm resin (Phenomenex, Torrance, CA). Peptides were eluted over a 12 min gradient from 1 % to 50 % acetonitrile, containing 0.1 % formic acid and at a flow rate of 800 nL/min. The eluted peptides were directed into the nanospray source of the mass spectrometer. All acquisition methods used the following parameters: a spray voltage of 2200 V, capillary temperature of 270°C, 10 ms of dwell time, Q1 FWHM of 0.2 and Q3 FWHM of 0.7. All study samples were processed in triplicate. Mass spectrometry data was analyzed by Pinpoint1.0 and/or Xcalibur2.1.

### Statistical methods

Area Under Curve (AUC) for the endogenous peptide and AUC of the isotopically labeled internal standard peptide were used to calculate peptide quantity and the data collated by Excel. The concentration of endogenous EGFR peptide that results from processing of cells/tissue with the Liquid Tissue protocol and reagents for each sample was calculated using the following formula:

(1) $$\frac{\text{AUCEndogenouspeptide}}{\text{AUC }\left(\text{isotopicallylabeledpeptide}\right)}\times \frac{\text{amol }\left(\text{isotopicallylabeledpeptide}\right)}{\mu \text{gproteinanalyzed}}=\text{amolEGFRpeptide per}\mu \text{goftissueprotein}$$

Quantitation of EGFR peptide was normalized across all samples based on the total amount of protein analyzed in a given sample, and not the number of cells analyzed. Cell counts were approximated but may not be counted accurately during tissue microdissection, thus the uncertainty and inability to quantitate total cellular content from FFPE. All correlation curves and linear regression values were developed in Excel for both ELISA immunoassay and SRM assay, and also for comparisons between ELISA and SRM.

## Results

### Immunoanalysis of non-fixed cells

Four (4) tissue culture cell lines suspected of expressing EGFR were analyzed by immunoassay to provide quantitative benchmarks to experimentally assess the ability of the mass spectrometry-based SRM assay for the EGFR protein (EGFR-SRM assay) to precisely reflect the amount of total EGFR protein per μg of total protein in formalin fixed biological samples. Cells were grown in culture, fresh protein lysates prepared, and ELISA-based sandwich immunoassay performed to develop quantitative data for the EGFR protein utilizing established methodology directly in known samples.

**Figure 1:**
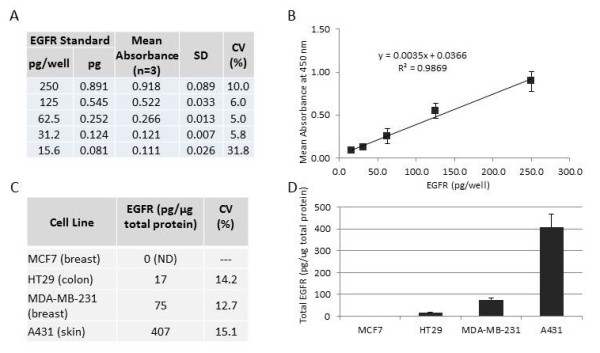
**Quantitative analysis of the epidermal growth factor receptor (EGFR) protein by enzyme-linked immunosorbent assay (ELISA) sandwich immunoassay in cultured cell lines as a basis for comparison with the mass spectrometry-based Selected Reaction Monitoring (SRM) analysis**. (**A**) Serial dilutions of a recombinant EGFR standard analyzed in triplicate by ELISA to establish a quantitative curve for comparison with EGFR levels from cell lines. (**B**) Plot of these data on a linear regression curve (R2 = 0.9869) (**C**) ELISA immunoanalysis of EGFR from protein lysates from non-fixed cells derived from 4 cell lines. (**D**) Plot of these data on a bar graph demonstrating a quantitative range of EGFR protein in the cell lines. CV = coefficient of variance.

A standard curve was prepared utilizing purified recombinant EGFR protein to allow for quantitation of EGFR. The EGFR standards were assayed in triplicate with good reproducibility (%CV range: 5.0 to 31.8) across serial dilutions that ranged in concentration from 15.6 pg/well (0.081 pg) in assay dilution buffer to 250 pg/well (0.891 pg) in assay dilution buffer (Figure 1A). The linear regression line was plotted as ELISA signal versus pg/well of EGFR protein standard and shows a high degree of linearity (R^2^ = 0.9869) indicating a curve against which ELISA assay data can be plotted to determine quantitative measure of the EGFR protein in lysates from the non-formalin fixed cultured cells (Figure 1B).

For analysis of the non-fixed cells, 0.1 μg of protein lysate from each of the 4 cell lines was analyzed in triplicate in the EGFR ELISA immunoassay and quantitative EGFR determined for each lysate by comparison to the standard curve. Results indicate a range of EGFR expression from not detected (ND) in MCF7 cells to 407 pg/μg total protein analyzed in A431 cells (Figure 1C). Bar graph analysis demonstrates the range of EGFR expression levels which provides for an excellent set of cell lines to compare SRM analysis of formalin fixed cells directly to this ELISA data of the matching non-fixed cell line preparations (Figure 1D).

These results demonstrate quantitative measurement of EGFR levels utilizing standard immunoassay across a collection of fresh, unfixed cell lines and establish a dataset that can be used to determine the ability of SRM methodology to measure the EGFR protein directly in the matching formalin fixed cells, and to demonstrate that SRM quantitation of a single EGFR peptide reflects the amount of total EGFR protein in formalin fixed cells.

### SRM assay development

**Figure 2:**
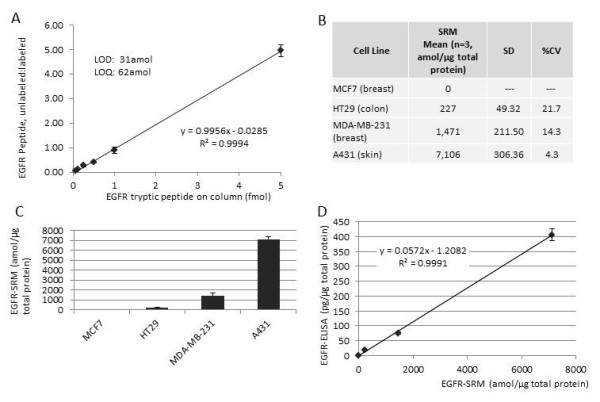
**Analysis of EGFR using mass spectrometry-based SRM (EGFR-SRM) of Liquid Tissue lysates from the formalin fixed paraffin embedded (FFPE) culture cells compared with ELISA analysis for EGFR from matching non-fixed cells**. (**A**) Standard SRM curve generated using unlabeled and isotopically labeled synthetic peptide that is used to define the limit of detection (LOD, 31 amol), define the limit of quantitation (LOQ, 62amol), and determine the linearity of the assay (R2 = 0.9994). (**B**) Triplicate EGFR-SRM analysis of Liquid Tissue lysates from formalin fixed cells. Quantitative data is reflected in amol/μg total protein. (**C**) Bar graph analysis of EGFR-SRM data. (**D**) Linear regression curve demonstrating correlation (R2 = 0.9991) between quantitative EGFR as determined by ELISA (EGFR-ELISA) analysis of non-fixed cells and quantitative EGFR-SRM analysis of the matching formalin fixed cells. SD = standard deviation.

The EGFR-SRM assay was developed in a stepwise fashion. Purified recombinant protein was trypsinized and peptides analyzed by Orbitrap and TSQVantage triplequadrupole mass spectrometry to evaluate all peptides and establish a list of candidates for absolute quantitation. Further narrowing of the candidates was achieved by focusing on candidate peptides in a TSQVantage analysis of a Liquid Tissue lysate prepared from formalin fixed EGFR-expressing A431 tissue culture cells. This stepwise process led to selection of a single EGFR tryptic peptide, IPLENLQIIR, which gave the best intensity in both trypsin digested recombinant EGFR and fixed A431 cells as well as reproducible transition ion ratios. The isotopically labeled internal standard synthetic peptide and the unlabeled synthetic peptide were used to build a standard curve against the background of the non-human, complex, tryptic peptide mixture from *P. furiosus*. The standard curve was developed such that the ratio of unlabeled:labeled peptides are plotted against increasing amounts of unlabeled peptide as measured against a constant 5fmol amount of the isotopically labeled internal standard peptide. Results indicate the limit of quantitation (LOQ) at 62amol on column, limit of detection (LOD) at 31amol on column, linear regression of R^2^ = 0.9994, and CVs ranging from 3.13 % to 15.81 % (Figure 2A). Achieving low attomolar LOD/LOQ values, tight linear regression, and minimal variation against a complex tryptic peptide mixture indicates development of a sensitive, highly reproducible, quantitative Liquid Tissue-SRM assay displaying 100% protein specificity for quantitation of this EGFR peptide in a complex biological sample*.*

### SRM analysis of fixed cells and correlation to immunoassay

The EGFR-SRM assay was performed on parallel cell cultures that were formalin fixed and paraffin embedded in order to establish the validity of the SRM assay and to demonstrate that this assay can precisely measure EGFR protein levels in formalin fixed samples. FFPE cell lines were processed in the same manner as FFPE tissue (microdissection and Liquid Tissue preparation) in order to replicate the steps involved in SRM analysis of FFPE tumor tissue. SRM analyses of FFPE cell lines were performed in triplicate and data was compared for quantitative purposes to the standard curve generated as described above (Figure 2A). Quantitation is reflected in attomoles of the IPLENLQIIR peptide detected per μg of total protein of Liquid Tissue lysate analyzed. Results demonstrate a range from ND in MCF7 cells to a high of 7,106amol/μg in A431 cells with a range in CVs from 4.3 % to 21.7 % (Figure 2B). As demonstrated by ELISA analysis of the non-fixed cultures, bar graph analysis of SRM data of the matched formalin fixed cells indicates a range of EGFR expression levels, suggesting high correlation of SRM analysis of formalin fixed cells to ELISA data from parallel-processed fresh, unfixed cells (Figure 2C).

Analytical comparison of the ELISA immunoassay to the SRM method was performed on a correlation curve. SRM data (amol/μg total protein) of formalin fixed cell lines were plotted against ELISA data (pg/μg total protein) of matching non-fixed culture cells. The response curve shows a high degree of correlation with a linear regression value of R^2^ = 0.9991 (Figure 2D). These results demonstrate the Liquid Tissue-SRM technology platform reproducibly quantitates levels of a single EGFR peptide in formalin fixed cells and that the measured levels are highly correlated to immunoassay of EGFR protein in matching cultures of non-fixed cells. Thus, the SRM assay of EGFR reliably and precisely reflects levels of the EGFR protein in formalin fixed cultured cells. Development of a reliable and reproducible quantitative assay for an EGFR peptide that accurately reflects EGFR protein levels in both non-fixed and formalin fixed biological samples strongly suggests quantitation of this peptide directly in FFPE tumor tissue will accurately reflect EGFR protein levels in clinical specimens of FFPE tumor tissue.

### SRM Analysis of Xenograft Tumors

A collection of FFPE human xenograft tumors of NSCLC origin (n = 10) were analyzed by the EGFR-SRM assay. Tissues from this collection display histological subtypes, different patterns of cellular differentiation, and varying levels of EGFR expression as previously determined by IHC of formalin fixed tumor tissue, western blot analysis of frozen matching tumor tissue, and SRM of frozen matching tissue thus providing a unique set of FFPE tumor samples with known EGFR status [21]. Xenograft tumors were sectioned onto DIRECTOR slides, tumor cells microdissected to isolate human tumor cells from mouse tissue, Liquid Tissue lysates prepared, and lysates analyzed by SRM in triplicate. Results indicate quantitation of a broad range of the EGFR peptide from 305amol/μg to 12,860amol/μg protein (>200X LOQ) with variation ranging from CV = 0.2 % to 40 % (Table 1). Comparing SRM data to the previous EGFR analyses indicates SRM results consistent with both western blot and SRM analysis of the matched frozen tumor tissue and show a correlative trend to IHC data of the FFPE tumors [21]. These results demonstrate SRM application directly in known EGFR-positive FFPE tumor tissue and further suggest capability to quantify the EGFR protein directly in FFPE tumor tissue.

**Table 1:** EGFR-SRM analysis of formalin-fixed paraffin-embedded (FFPE) human xenograft tissue of non-small cell lung carcinoma (NSCLC) origin

**Sample**	**Histology Subtype**	**Cellular Differentiation**	**EGFRSRM (amo/μg total protein)**	**CV%**
ADC1	Adeno	Moderate	2,113	9.6
ADC2	Adeno	Poor	3,378	4.6
ADC3	Adeno	Moderate	5,591	9.6
ADC4	Adeno	Poor	305	10.9
ADC5	Adeno	Poor	312	9.2
SCC1	Squamous	Well	467	0.2
SCC2	Squamous	Well	826	0.2
SCC3	Squamous	Moderate	12,860	0.3
SCC4	Squamous	Poor	757	40
SCC5	Squamous	Moderate	1,736	1.4

Results are standardized to total amount of protein. SRM analysis is consistent with previously published western blot, immunohistochemistry (IHC), and SRM analysis of the matched frozen xenograft tissue [21].

### SRM analysis of NSCLC patient tissue

**Figure 3:**
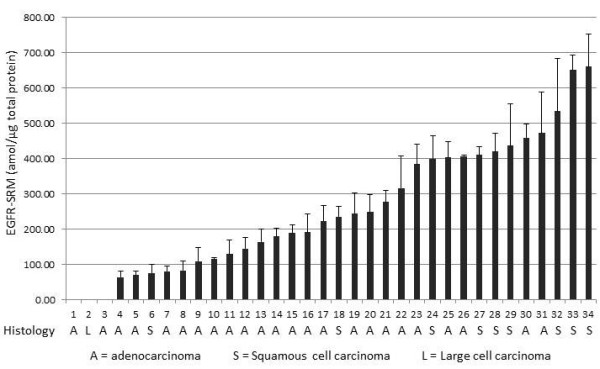
**SRM analysis for EGFR in FFPE tumor specimens taken from patients with non-small cell lung carcinoma (NSCLC) after treatment with the EGFR inhibitor gefitinib in the neoadjuvant setting**. SRM analysis across a cohort of formalin fixed tumor specimens demonstrates EGFR quantitation across a broad range of EGFR expression levels after treatment with gefitinib.

A collection of twenty-three (23) NSCLC patient tumor tissue samples from a phase II clinical trial was analyzed by the EGFR-SRM assay in order to demonstrate clinical validation of the assay directly in resected tissue from patients treated with an EGFR inhibitor and whose tumors are historically driven by the EGFR protein. Tissues were sectioned, cancerous cells microdissected, Liquid Tissue lysates prepared, and lysates analyzed in triplicate by SRM. Results demonstrate various quantitative levels of the EGFR peptide in patient tumor tissue samples with a broad range from ND to 660.73amol/μg (10X LOQ) with variation ranging from CV = 0.77 % to 35.38 % (Figure 3). Automated quantitation of three (3) tumor samples showed levels of EGFR which were below the LOQ as defined by the standard curve in *P. furiosus* complex proteomic matrix. Analysis of these patient samples demonstrates a large dynamic range within patient tissue suggesting real potential for this assay to be clinically useful. These results demonstrate measurement of EGFR directly in patient tumor tissue utilizing an assay that has demonstrated ability to accurately measure EGFR protein levels in formalin fixed tissue for use in improving clinical decisions about EGFR-targeted therapy.

## Discussion

This report describes the development of a highly sensitive mass spectrometry-based Selected Reaction Monitoring (SRM) assay capable of quantifying an important cancer drug target directly in formalin fixed paraffin embedded (FFPE) patient tumor tissue in a linear fashion across a large range of concentrations. In conjunction with standard pathological approaches, this assay can be used as a complementary or companion diagnostic to analyze clinical tumor specimens to support development of novel cancer therapies or to optimize patient selection for therapies currently in use. Companion diagnostics facilitate identification of patients likely to respond to drugs based on individual molecular profiles which allow customization of treatment, and thus, minimization of the risk of adverse reactions while maximizing the effect of properly administered drugs. Examples of successful therapeutics linked to companion diagnostic tests include Herceptin and Gleevec [23,24].

The EGFR protein is an important therapeutic target for cancer. Several FDA-approved therapeutic agents target the EGFR protein. A routine, standardized clinical method, however, has not yet been implemented, which can stratify patients by quantitative EGFR protein expression levels in relation to a projected benefit to EGFR-targeted therapies. The first step on this path is to start to precisely and reliably quantitate tumor expression of EGFR as a means of determining whose tumors express therapeutically-responsive levels of the EGFR protein.

Currently accepted methodology to analyze protein expression in standard, formalin fixed patient tissue is limited to immunohistochemistry (IHC), which provides visual cellular context, but suffers from numerous drawbacks including antibody specificity, low sensitivity, lack of objective quantitation, inter-laboratory methodology/analytical variability, lack of inter-laboratory standardization, and inability to multiplex biomarker analysis for high content analysis [25-30]. As such, a protein assay technology platform for application to formalin fixed patient tissue that overcomes these limitations is warranted. Mass spectrometry, and specifically selected reaction monitoring method as applied by triplequadrupole instrumentation, can provide for protein assays with high sensitivity, absolute specificity, objective quantitation, and multiplex capabilities [8-12]. The Liquid Tissue-SRM diagnostic technology platform utilizes selected reaction monitoring methodology to develop tissue diagnostic assays for use directly on formalin fixed patient tissue. The key to this platform is the Liquid Tissue protocol allowing FFPE tissue to be completely solublized resulting in a complex mixture of protease-digested peptides suitable for quantitative mass spectrometry [13,14].

Protein matter within tissue is preserved by formalin fixation due to the fact that intra- and intermolecular crosslinks are formed by formalin treatment effectively stabilizing all proteins, suspending enzymatic activity and preventing destruction [31]. Further, embedding in paraffin effectively removes all water from the tissue thereby allowing the tissue to be stored at room temperature for years with minimal deterioration. It has been shown, however, in a model system based on formalin fixation that enzyme activity and antigenicity of RNAse can be restored by extensive heating showing that this protein has been well preserved within formalin fixed tissue, and that widespread protein deterioration has not occurred [32-34]. Formalin fixation also reduces antigenicity of proteins within tissue due to cross linking and this is an important consideration when using antibodies to detect proteins in fixed tissues. It should be noted that optimization of antigen retrieval is essential to utilize antibody-based methods to identify proteins from formalin fixed tissue. This approach is especially important for standardizing immunohistochemical methods across labs and for attempting to multiplex IHC in fixed tissue.

The Liquid Tissue process is based on heat retrieval of proteins present in tissue followed by protease digestion. Placing formalin fixed tissue/cells in a buffer followed by extended heating at elevated temperature has two effects: 1) reversal of formalin-induced crosslinks, and 2) removal of secondary/tertiary/quaternary protein structure to allow for protease digestion of the primary protein structure. While obtaining information from the expanded protein structure is important in many studies, the Liquid Tissue process is deducing information from the primary protein structure by analyzing peptide fragments only, and any lack of information about secondary/tertiary/quaternary structure does not impede the powerful advancement that the Liquid Tissue protocol and reagents afford. This methodology utilizes patient tissue that has been prepared using standard histopathology methodology and, as such, represents a powerful advance translatable to clinical research.

Multiple reports have demonstrated mass spectrometric analysis of formalin fixed tissue and these studies show comparable results are obtained between formalin fixed and matching frozen tissue [14,35-38]. When combined with tissue microdissection for collection of specific diseased cell populations the resulting Liquid Tissue lysate represents the molecular histology/profile of the disease where expression of proteins associated with the disease process can be monitored.

This platform was used to develop a quantitative assay for the EGFR protein in FFPE tissue samples taken from patients with NSCLC enrolled in a phase II neoadjuvant trial of the EGFR inhibitor, gefitinib. Assay performance was demonstrated on cell culture models of formalin fixed tissue indicating the ability to accurately measure levels of the EGFR protein directly in formalin fixed samples across a wide range of EGFR concentrations. The reliability of this analytical approach was demonstrated by application of this assay to FFPE human xenograft tumor tissue, in which the ability to measure EGFR protein levels was demonstrated to be consistent with previously-determined EGFR levels in these samples. The application of this assay to a cohort of FFPE tissues surgically resected from NSCLC patients that participated in an EGFR-targeted therapeutic trial indicates its ability to monitor EGFR levels directly in patient-derived tumor tissue.

Potential applications of this assay include the ability to identify cancer patients that would benefit from EGFR-inhibitor therapies and may indicate a mode of drug resistance for growth factor receptor-targeted therapies where a “kinase switch” mechanism for acquired resistance to IGF-1R and cMet inhibition results from co-expression of EGFR in the cancer cells. Combining the current EGFR assay in a multiplex fashion with similar assays for IGF-1R and cMet could provide for optimal therapeutic choice and a drug resistance biomarker panel to drive targeted therapeutic efforts in NSCLC, as well as other cancers.

This EGFR-SRM assay represents a highly promising approach to quantitating and monitoring EGFR levels in patient tumor tissue. Immuno-based methods, such as western blot and ELISA, cannot be effectively performed on FFPE patient tissue due to formalin-induced crosslinking, and IHC methods struggle to achieve specificity, reliability and reproducibility. Reports have demonstrated analysis of protein from formalin fixed tissue using western blot. These approaches, however, suffer from lack of total representation because all protein is not solubilized prior to gel separation and the majority of proteins detected by western blot are limited to highly abundant proteins [39,40]. Because the Liquid Tissue protocol generates protease-digested peptides from formalin fixed tissue, it cannot be used for preparing sample for ELISA and/or western blot.

SRM methodology provides a quantitative, sensitive, and reproducible approach for analysis of proteins in any biological sample and this report demonstrates the application of SRM methodology to measuring EGFR protein levels in FFPE biological samples, including patient tumor tissue. Experiments to establish clinical utility of this assay for patient stratification, choice of therapy, and drug resistance prediction are ongoing and will be an important next step in developing companion diagnostic assays for EGFR-targeted therapy.

## Abbreviations

ACN: Acetonitrile; AUC: Area under the curve; DMEM: Dulbecco’s Modified Eagle Medium; EGFR: Epidermal growth factor receptor; EGFR-ELISA: Epidermal growth factor receptor as determined by enzyme-linked immunosorbent assay; EGFR-SRM: Selected Reaction Monitoring assay for epidermal growth factor receptor; ELISA: Enzyme-linked immunosorbent assay; FBS: Fetal bovine serum; FFPE: Formalin fixed paraffin embedded; IHC: Immunohistochemistry; LC: Liquid chromatography; LOD: Limit of detection; LOQ: Limit of quantitation; MS: Mass spectrometry; NBF: Neutral-buffered formalin; ND: Not detected; SRM: Selected Reaction Monitoring; NSCLC: Non-small cell lung cancer..

## Competing interests

Authors declare that they have no competing interests.

## Authors’ contributions

TH: Designed experiments, Performed experiments, Analyzed the data. ST: Designed experiments, Performed experiments, Analyzed the data. WLL: Designed experiments, Performed experiments, Analyzed the data. MMD: Performed experiments, Analyzed the data. JA: Performed experiments. KMB: Performed experiments. PT: Performed experiments. JT: Performed experiments. HLG: Contributed samples. TKW: Contributed samples. MFM: Designed experiments. MST: Contributed samples. DBK: Designed experiments, Analyzed the data, Wrote the manuscript. Jon Burrows: Designed experiments, Funded the experiments. All authors approved the final manuscript.
